# Overexpression of TPL2 may be a predictor of good prognosis in patients with breast invasive ductal carcinoma

**DOI:** 10.1038/s41598-023-44660-z

**Published:** 2023-10-13

**Authors:** Qin Yu, Dan Wan, Rong Fu, Feng Li, Yutao Zhang

**Affiliations:** Department of Pathology, The First People’s Hospital of Zigong, Zigong, 643099 China

**Keywords:** Cancer, Biomarkers, Oncology

## Abstract

The objective of this study was to investigate the clinical significance and roles of tumor progression locus 2 (TPL2) and peptidyl-prolyl cis–trans isomerase 1 (Pin1) in the occurrence and development of breast invasive ductal carcinoma (IDC). Immunohistochemistry was used to detect the expression of TPL2 and Pin1 in human breast tissues, which included normal breast tissues (Normal), tissues with fibrocystic changes (FCC), ductal carcinoma in situ (DCIS), and IDC. The roles of TPL2 and Pin1 in the occurrence and development of IDC, as well as the correlation between their expression levels and clinicopathological parameters, were analyzed. Compared with Normal and FCC groups, the overexpression of TPL2 and Pin1 was significantly increased in DCIS and IDC groups (DCIS vs Normal: *P* = 0.002/*P* < 0.001; IDC vs Normal: *P* = 0.007/*P* = 0.003; DCIS vs. FCC: *P* = 0.008/*P* = 0.004; IDC vs. FCC: *P* = 0.04/*P* = 0.043). The expression levels of TPL2 and Pin1 were positively correlated in DCIS and IDC groups (*P* = 0.001, *P* = 0.011). In the IDC group, the Ki67 level in the TPL2 overexpression group was significantly lower than that in the TPL2 low expression group (*P* = 0.02). The TPL2 overexpression rate was significantly higher in IDC with histological grades 1–2 than that in IDC with histological grade 3 (*P* = 0.029). The TPL2 overexpression rate in IDC with tumor-node-metastasis (TNM) stage I was significantly higher than that in IDC with TNM stages II–III (*P* = 0.035). We conclude that TPL2 and Pin1 may synergistically promote the occurrence and development of IDC, but TPL2 overexpression may be an early molecular event in IDC development. TPL2 overexpression is significantly related with IDC with lower malignancy or earlier TNM stage, suggesting that the prognosis of IDC patients with TPL2 overexpression may be better and TPL2 overexpression may be a predictor of good prognosis in IDC.

## Introduction

Breast cancer is the most common malignant tumor in women worldwide and the most common cancer-related cause of death in women, and its incidence is increasing annually^1,2^. The pathogenesis of breast cancer has not been completely elucidated because it involves a variety of factors, genes, and signaling pathways.

Activation of mitogen-activated protein kinases (MAPKs) is closely related to oncogenic events. Tumor progression locus 2 (TPL2), also known as mitogen-activated protein kinase kinase kinase 8 (MAP3K8) or cancer Osaka thyroid oncogene (Cot), is an important tertiary serine/threonine protein kinase of the MAPK family, which plays important roles in the regulation of cell proliferation, differentiation, and apoptosis^3^. Several investigations have reported that TPL2 is involved in the occurrence and development of tumors and is abnormally expressed in a variety of malignancies^4–6^. At present, research on TPL2 is gaining momentum, and a few studies have reported that TPL2 is overexpressed in human breast cancer^7,8^. However, the oncogenic mechanism of TPL2 is still unclear.

Peptidyl-prolyl cis–trans isomerase 1 (Pin1) is a highly conserved polypeptide prolyl cis–trans isomerase. Pin1 specifically catalyzes the cis–trans isomerization of the phosphorylated serine/threonine-proline motif (pSer/Thr-Pro)^9^, thereby regulating protein conformation and activating multiple signaling pathways in tumorigenesis^10^. Previous studies have reported that Pin1 plays key roles in cell proliferation and transformation. It can also promote the occurrence and development of breast cancer^11–13^.

A previous breast cancer study has reported that TPL2 facilitates tumorigenesis by phosphorylating Pin1^8^. However, few studies have investigated the roles of these two molecules in the development of breast cancer. In addition, the sample size of previous studies was small, and the maximum sample size of breast cancer was only 40 cases^14^. More importantly, the correlation between TPL2 expression, clinicopathological parameters, and prognosis of patients with breast cancer has not been reported in the literature. Therefore, it is important to increase the sample size to further investigate the roles of TPL2 and Pin1 in the occurrence and development of breast cancer and their effects on treatment and prognosis.

According to the World Health Organization Breast Tumor Classification, the most common histological type of breast cancer is invasive ductal carcinoma (IDC)^15^. Previous studies on the roles of TPL2 and Pin1 in the development of IDC have only involved normal breast tissue and breast IDC tissue, but not precancerous lesions of IDC, namely ductal carcinoma in situ (DCIS). However, it is undeniable that including both precancerous lesions and IDC in the study would generate more convincing results. In addition, it is well known that fibrocystic changes (FCC) are common lesions of the breast^16^. Mammary duct epithelial hyperplasia is a common manifestation of FCC, and IDC is often accompanied by FCC, but it is still unclear whether FCC is a precursor lesion and a high-risk factor for IDC^17^. In this study, Normal, FCC, DCIS, and IDC were included for the first time to examine the expression of TPL2 and Pin1 in these groups, and to analyze the correlation between the two molecules and clinicopathological parameters in patients with IDC. On the one hand, the relationship between the above two molecules and the pathogenesis, treatment, and prognosis of IDC was discussed. On the other hand, whether FCC is a precursor lesion and a high-risk factor for IDC was also explored.

## Materials and methods

### Patients and tissue samples

This study was conducted with the approval of the Institutional Review Board of the First People’s Hospital of Zigong (No. 20190040), and written informed consent was obtained from all patients or their families. All methods were performed in accordance with the relevant guidelines and regulations. One hundred and seventy breast aspiration biopsy specimens and surgical resection specimens diagnosed by pathological examination at the Department of Pathology of the First People’s Hospital of Zigong from March 2017 to April 2020 were collected, including 30 cases of normal breast tissues (Normal), 30 cases of FCC, 30 cases of DCIS, and 80 cases of IDC. All patients were female, aged 26–77 years, with a median age of 49 years and a mean age of 50.4 years. No other benign breast lesions, such as fibroadenoma, were present in FCC patients. No patient with DCIS or IDC had other breast tumors, and no patient received antitumor therapy before surgery.

### Methods

#### Immunohistochemistry

An automatic immunohistochemical staining system (BenchMark GX; Ventana Medical Systems, Inc., Tucson, AZ, USA) was used for immunohistochemistry. Known sections of tissues were used as the positive control (Table 1), whereas phosphate-buffered saline (PBS) was used in place of the primary antibody as the blank control. Information on primary antibodies is provided in Table 1.

**Table 1:** Primary antibodies used in this study.

Antibody	Manufacturer	Clone or product ID	Dilution ratio	Expression	Positive control
MAP3K8	Abcam, UK	ab137589	1:500	Cytoplasm	Rectal adenocarcinoma
Pin1	Santa Cruz Biotechnology, USA	sc-46660	1:100	Nucleus and cytoplasm	Benign prostatic hyperplasia
Ki67	Maixin, China	kit-0005	Ready-to-use	Nucleus	Lymph node
ER	Maixin, China	kit-0012	Ready-to-use	Nucleus	Endometrioid carcinoma
PR	Maixin, China	kit-0013	Ready-to-use	Nucleus	Endometrioid carcinoma

MAP3K8, mitogen-activated protein kinase kinase kinase 8; Pin1, peptidyl-prolyl cis–trans isomerase 1; ER, estrogen receptor; PR, progesterone receptor.

#### Interpretation of immunohistochemistry results

The immunohistochemical staining results of TPL2 and Pin1 were evaluated by a semi-quantitative method. Semi-quantitative results were expressed as the histochemistry score (H-score), a widely used semi-quantitative method for immunohistochemical staining results. The H-score is based on the staining intensity (0, 1 + , 2 + , 3 +) and the percentage of positive cells (0–100%). Cell staining intensity was evaluated as follows: 0 indicated no staining; 1 + indicated pale yellow staining; 2 + indicated yellow staining; 3 + indicated yellow–brown staining. The H-score was calculated using the following formula: (% negative cells × 0) + (%1 + cells × 1) + (%2 + cells × 2) + (%3 + cells × 3). The H-score ranged from 0 to 300, with higher scores indicating higher protein expression. All cases were independently scored by two experienced pathologists, and the final H-score for each case was obtained from the average of the H-scores of both pathologists. Cases with H-score differences greater than 5 points were simultaneously reviewed by both pathologists, and a consensus score was reached. Overexpression of TPL2 and Pin1 was defined as H-score ≥ 150.

The immunohistochemical results of Ki67 were interpreted as follows: tumor cells with nuclear staining were defined as showing positive Ki67 expression. The proportion of Ki67 positive tumor cells in all tumor cells was defined as the Ki67 positive index. All cases were independently evaluated by two experienced pathologists.

According to the Estrogen and Progesterone Receptor Testing in Breast Cancer: ASCO/CAP Guideline Update (2020 edition)^18^, the immunohistochemical results of estrogen receptor (ER) and progesterone receptor (PR) were interpreted as follows: negative indicated that < 1% of tumor cells showed nuclear staining; low positive (expression levels of positive controls should be reported and comments should be added: patients were recommended for hormone-based treatment) indicated that 1–10% of tumor cells showed nuclear staining, and positive indicated that > 10% of tumor cells showed nuclear staining, that is, ≥ 1% of tumor cell nuclei were defined as showing positive ER or PR expression. All cases were independently evaluated by two experienced pathologists. Immunohistochemical sections were observed under an Olympus BX46 optical microscope (Olympus Corp., Tokyo, Japan). Images were captured with an Olympus DP27 microscopic imaging system (Olympus Corp.).

#### Clinicopathological parameters of IDC cases

Histological grading of all IDC cases was conducted according to Elston and Ellis’ Nottingham Modification of the Scarff-Bloom-Richardson method^19^. Tumor-node-metastasis (TNM) staging of all IDC cases was conducted according to the American Joint Committee on Cancer Staging Manual (8^th^ edition)^20^.

#### Statistical analysis

Statistical analyses were performed using SPSS 21.0 statistical software (IBM, Armonk, NY, USA). Differences between two groups were compared using Pearson’s chi-square test or independent samples* t*-test. Correlation analysis was performed using Fisher’s exact test or Pearson’s chi-square test. *P* values less than 0.05 were considered statistically significant.

## Results

### Expression analysis of TPL2 and Pin1 in different breast tissues

**Figure 1:**
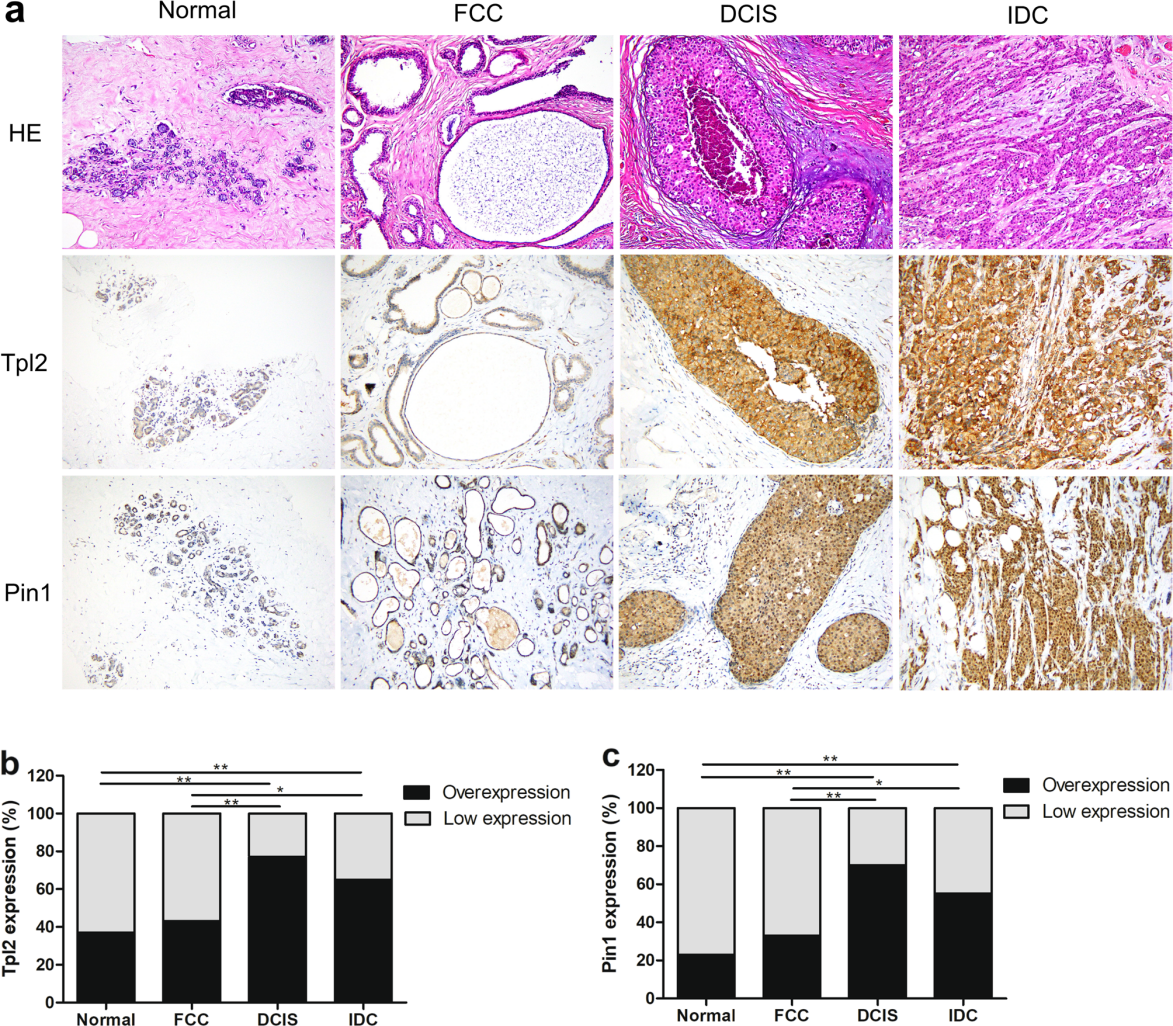
Analysis of TPL2 and Pin1 expression in Normal, FCC, DCIS, and IDC groups. (**a**) Representative images show the immunohistochemical staining of TPL2 and Pin1 and the hematoxylin–eosin (HE) staining in different breast tissues (magnification, ×200). (**b**) Comparison of TPL2 overexpression rates in different breast tissues. **c** Comparison of Pin1 overexpression rates in different breast tissues. Statistical analysis was performed using Pearson’s chi-square test, *P < 0.05, **P < 0.01. TPL2: tumor progression locus 2. Pin1: peptidyl-prolyl cis–trans isomerase 1. HE, hematoxylin–eosin; FCC, fibrocystic changes; DCIS, ductal carcinoma in situ; IDC, invasive ductal carcinoma.

Immunohistochemistry was used to detect the expression status of TPL2 and Pin1 in the Normal group (n = 30), FCC group (n = 30), DCIS group (n = 30), and IDC group (n = 80). The results revealed that the TPL2 overexpression rates in Normal, FCC, DCIS, and IDC groups were 36.7% (11/30), 43.3% (13/30), 76.7% (23/30), and 65% (52/80), respectively. Compared with Normal and FCC groups, TPL2 expression levels were significantly increased in DCIS and IDC groups (Fig. 1a, b, DCIS vs Normal: *P* = 0.002; IDC vs Normal: *P* = 0.007; DCIS vs FCC: *P* = 0.008; IDC vs FCC: *P* = 0.04), but there were no significant differences between the FCC group and the Normal group, and also between the DCIS group and the IDC group (Fig. 1b, FCC vs Normal: *P* = 0.598; DCIS vs IDC: *P* = 0.242).

The expression status of Pin1 in each group was similar to that of TPL2. The Pin1 overexpression rates in Normal, FCC, DCIS and IDC groups were 23.3% (7/30), 33.3% (10/30), 70% (21/30), and 55% (44/80), respectively. Compared with Normal and FCC groups, Pin1 expression was significantly increased in DCIS and IDC groups (Fig. 1a, c, DCIS vs Normal: *P* < 0.001; IDC vs Normal: *P* = 0.003; DCIS vs FCC: *P* = 0.004; IDC vs FCC: *P* = 0.043), but there were no significant differences between the FCC group and the Normal group, and also between the DCIS group and the IDC group (Fig. 1c, FCC vs Normal: *P* = 0.39, DCIS vs IDC: *P* = 0.154). The results showed that TPL2 and Pin1 were overexpressed in both DCIS and IDC groups, but there were no increasing or decreasing expression trends in Normal, FCC, DCIS, and IDC groups.

### Correlation analysis of Pin1 and TPL2 expression in DCIS and IDC cases

**Figure 2:**
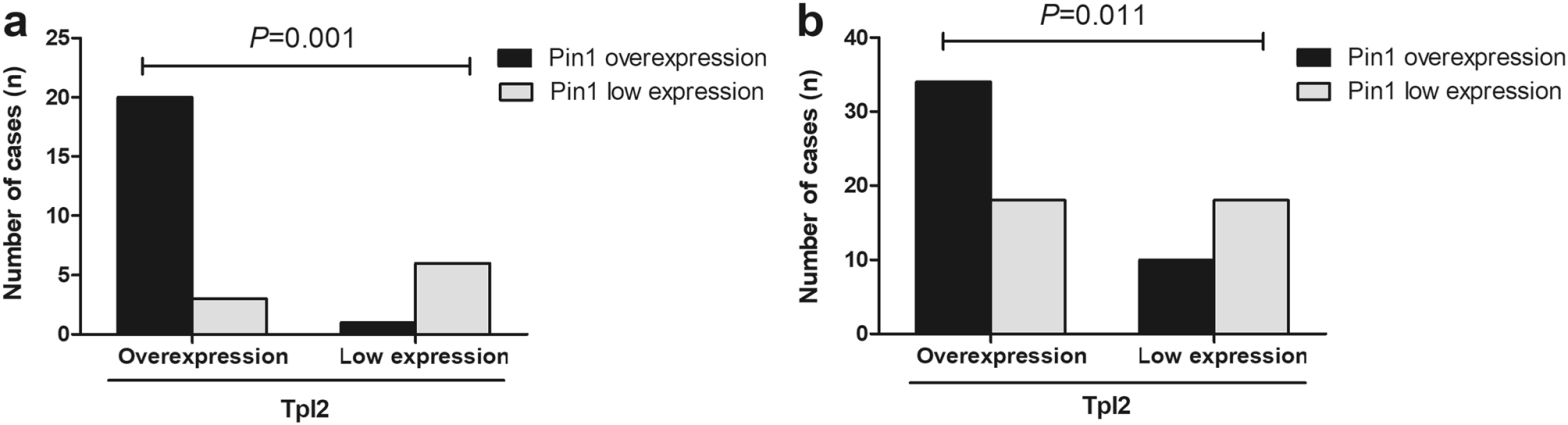
Correlation analysis of TPL2 and Pin1 expression in DCIS and IDC cases. (**a**) Correlation analysis of TPL2 and Pin1 expression in the DCIS group using Fisher’s exact test for statistical analysis (*P* = 0.001). (**b**) Correlation analysis of TPL2 and Pin1 expression in the IDC group using Pearson’s chi-square test for statistical analysis (*P* = 0.011). TPL2, tumor progression locus 2; Pin1, peptidyl-prolyl cis–trans isomerase 1; DCIS, ductal carcinoma in situ; IDC, invasive ductal carcinoma.

Among the 23 DCIS cases with TPL2 overexpression, there were 20 cases with Pin1 overexpression. Among the 7 DCIS cases with low TPL2 expression, 6 cases also showed low Pin1 expression (Fig. 2a, *P* = 0.001). On the other hand, among the 52 IDC cases with TPL2 overexpression, there were 34 cases with Pin1 overexpression. Among the 28 IDC cases with low TPL2 expression, 18 cases also showed low Pin1 expression (Fig. 2b, *P* = 0.011). The results revealed that the expression of TPL2 and Pin1 was positively correlated in DCIS and IDC groups, suggesting that TPL2 and Pin1 may synergistically promote the occurrence and development of IDC of the breast.

### Relationship between expression levels of TPL2 and Pin1 and clinicopathological parameters in IDC cases

**Figure 3:**
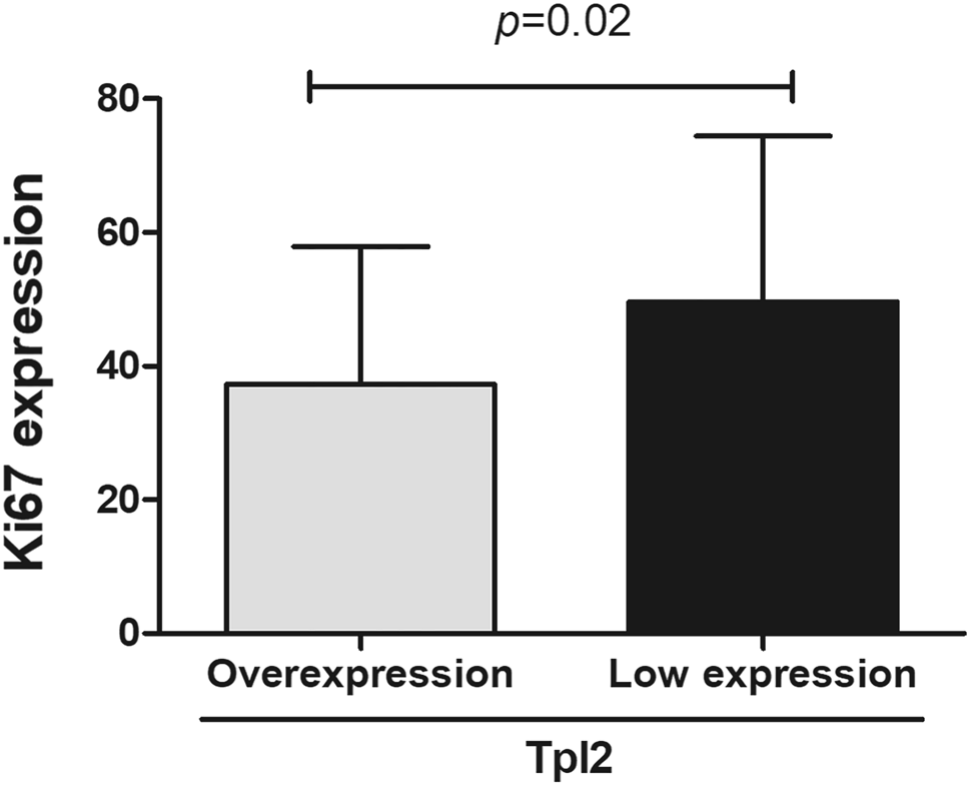
Comparison of the Ki67 expression status in IDC cases with TPL2 overexpression and low expression. Statistical analysis was performed using the independent samples *t*-test (*P* = 0.02). TPL2, tumor progression locus 2.

The relationships between TPL2 and Pin1 expression with patient age, Ki67 positive index, histological grading, tumor size, lymph node metastasis, TNM stage, and positive ER/PR expression were analyzed in 80 IDC cases. The results showed that the Ki67 positive index in the high TPL2 expression group was significantly lower than that in the low TPL2 expression group (Fig. 3, *P* = 0.02). Furthermore, the TPL2 overexpression rate in IDC cases with histological grading 1–2 was 74% (37/50), whereas the TPL2 overexpression rate in IDC cases with histological grading 3 was 50% (15/30), indicating that the overexpression rate of TPL2 in IDC grades 1–2 was significantly higher than that in IDC grade 3 (Table 2, *P* = 0.029). In addition, TNM staging of all IDC cases was conducted according to the American Joint Committee on Cancer Staging Manual (8^th^ edition)^20^. The results revealed that the TPL2 overexpression rate in stage-1 IDC cases was 87.5% (14/16), whereas the TPL2 overexpression rate in stage-II–III IDC cases was 59.4% (38/64). These findings indicate that the TPL2 overexpression rate in stage-I IDC was significantly higher than that in stage-II–III IDC (Table 2, *P* = 0.035). We also found that the Pin1 overexpression rate in IDC cases with ER positive expression was 65.3% (32/49), but the Pin1 overexpression rate in IDC cases with negative ER expression was 38.7% (12/31), that is, the Pin1 overexpression rate of the ER positive expression group was significantly higher than that of the ER negative expression group (Table 2, *P* = 0.02). The remaining data showed that TPL2 expression was not correlated with lymph node metastasis, patient age, and tumor size, and Pin1 expression was also not correlated with the other clinicopathological parameters (Table 2).

**Table 2:** Relationships between TPL2 and Pin1 expression with clinicopathological parameters in IDC.

	n	TPL2			Pin1		
		≥ 150	χ2	*P*	≥ 150	χ2	*P*
Age							
< 50 years	31	19 (61.3%)	0.306	0.58	16 (51.6%)	0.235	0.628
≥ 50 years	49	33 (67.3%)			28 (57.1%)		
Tumor size							
≤ 2 cm	26	20 (76.9%)	2.407	0.121	16 (61.5%)	0.665	0.415
> 2 cm	54	32 (59.3%)			28 (51.9%)		
Histological grade							
1–2	50	37 (74%)	4.747	**0.029**	30 (60%)	1.347	0.246
3	30	15 (50%)			14 (46.7%)		
Lymph node metastasis							
Yes	48	29 (60.4%)	1.108	0.293	24 (50%)	1.212	0.271
No	32	23 (71.9%)			20 (62.5%)		
TNM stage							
I	16	14 (87.5%)	4.451	**0.035**	11 (68.8%)	1.528	0.216
II–III	64	38 (59.4%)			33 (51.6%)		
ER							
+	49	35 (71.4%)	2.297	0.13	32 (65.3%)	5.427	**0.02**
-	31	17 (54.8%)			12 (38.7%)		
PR							
+	37	27 (73.0%)	1.923	0.165	24 (64.9%)	2.707	0.100
-	43	25 (58.1%)			20 (46.5%)		

TPL2, tumor progression locus 2; Pin1, peptidyl-prolyl cis–trans isomerase 1; TNM, tumor-node-metastasis; ER, estrogen receptor; PR, progesterone receptor.

*Pearson’s chi-square test.

Significant are in value bold.

### Follow-up data of IDC cases

There was a total of 80 IDC cases, with 2 patients not responding to telephone interviews and 78 patients successfully followed up. After 3 years and 9 months to 5 years of observation, 70 patients were still alive; the survival rate was 89.7%, which was consistent with the 5-year survival rate and good overall prognosis of breast cancer. As the proportion of censored data is too large (70/78), it would have substantially affected the results of survival analysis; thus, survival analysis could not be conducted.

## Discussion

TPL2, an important tertiary serine/threonine protein kinase of the MAPK family, consists of a serine/threonine kinase domain, an amino-terminal region of unknown function, and a carboxy-terminal region that contains a phosphorylation site and determines protein stability^3,21^. As a potent kinase with broad substrate specificity, TPL2 can regulate various signaling pathways to modulate cell proliferation, apoptosis, and metastasis, and it also plays a dual role in tumor promotion and tumor suppression in the occurrence and development of a variety of tumors^4–6,22,23^. Previous studies have reported that overexpression of TPL2 can promote the development of breast cancer^7,8,14^, consistent with our findings. In this study, we observed that the TPL2 overexpression rate was significantly higher in DCIS (23/30, 76.7%) and IDC (52/80, 65%) groups than that in the Normal group (11/30, 36.7%), which indicated that TPL2 was not only overexpressed in IDC but also in DCIS, suggesting that TPL2 plays a role in promoting the development of breast cancer.

The occurrence and development of breast cancer involves complex pathological processes with multiple factors and several stages, which require the participation of a variety of related molecules. A common biological feature of malignant tumors is uncontrolled cell proliferation. A key signaling mechanism that controls cell proliferation and transformation is protein phosphorylation. As a peptidyl prolyl cis–trans isomerase, Pin1 is a key molecule that mediates the progression of multiple malignancies^9,20^, and it regulates the conformation of many proteins by catalyzing the cis–trans isomerization of phosphorylated protein pSer/Thr-Pro, thereby inducing cell proliferation and malignant transformation. Previous studies have reported that Pin1 promotes the oncogenic program of breast cancer through multiple mechanisms and is overexpressed in breast cancer cell lines and tissues^11–13,24^. The data of this study showed that the Pin1 overexpression rate in DCIS (21/30, 70%) and IDC (44/80, 55%) groups was significantly higher than that in the Normal group (7/30, 23.3%), indicating that Pin1 is overexpressed in human breast cancer tissues.

Undeniably, the relationship between FCC and IDC is complicated due to the frequent presence of mammary duct epithelial hyperplasia, which is a manifestation of FCC, and it is unclear whether FCC is a precursor lesion and a high-risk factor of IDC^17,25^. Therefore, we included the FCC group into our analysis, and investigated the expression differences of TPL2 and Pin1 between the FCC group and the other three groups. The TPL2 overexpression rate in the FCC group was 43.3% (13/30), which was not statistically different from that of the Normal group, but it was significantly lower than those of DCIS and IDC groups. The same approach was used to analyze the Pin1 overexpression rate in the four groups, and the results were similar to those of TPL2. TPL2 and Pin1 were overexpressed in both DCIS and IDC groups, but not in the FCC group. Therefore, we speculate that FCC is not a precursor lesion and a high-risk factor for IDC.

To further investigate whether TPL2 and Pin1 have synergistic effects in promoting the occurrence and development of breast cancer, the expression status of those two proteins in DCIS and IDC groups was studied. The results showed that expression of TPL2 and Pin1 was positively correlated in DCIS and IDC groups. Pin1 is also overexpressed in most tumors with high TPL2 expression, suggesting that TPL2 and Pin1 may synergistically promote the occurrence and development of breast cancer. For example, a study by Kim et al. demonstrated that the expression of TPL2 and Pin1 is positively correlated in human breast cancer tissues^8,14^. The study revealed that TPL2 induces the phosphorylation of Pin1 and increases the expression of cyclin D1, thereby promoting the occurrence of breast cancer^8^. In addition, the authors demonstrated that interleukin-22 promotes epithelial cell transformation and breast cancer development by increasing TPL2 phosphorylation and subsequently activating MEK–ERK, JNK–c-Jun, and STAT3 signaling pathways, and Pin1 was identified as a major positive regulator of these signaling pathways^14^. These findings show that the mechanism of TPL2 and Pin1 in promoting the occurrence and development of breast cancer is very complex, involving many signaling molecules and pathways. Presently, there are few studies on the mechanisms of action of TPL2 and Pin1 in the occurrence and development of breast cancer. Although our study pointed out that TPL2 and Pin1 may synergistically promote the occurrence of breast cancer, the specific mechanisms of action have not yet been elucidated. Therefore, it is necessary to conduct in-depth research on this in the future and identify new therapeutic targets for breast cancer.

The prognosis of IDC is closely related to tumor histological grade and TNM stage. In addition, as a reliable marker of tumor cell proliferation, Ki67 can also reflect the malignancy and prognosis of IDC to a certain extent. In IDC, a higher Ki67 positive expression index predicted a higher malignancy degree and a worse prognosis^26,27^. To investigate the relationship between TPL2 and Ki67 expression status, 80 cases of IDC were divided into the TPL2 overexpression group and the TPL2 low expression group, and the Ki67 expression level in the two groups was compared and analyzed. The results showed that the Ki67 expression level in the TPL2 overexpression group was significantly lower than that in the TPL2 low expression group. This study also found that the TPL2 overexpression rate in IDC with histological grades 1–2 was significantly higher than that in IDC with histological grade 3. The correlation between TPL2 expression and TNM stage was also analyzed, and the results showed that the TPL2 overexpression rate of IDC in stage I was significantly higher than that of IDC in stages II–III. Similar findings were also reported by Sourvinos et al., who found a significant association between TPL2 overexpression and stage I breast cancer^7^.

The above-mentioned three studies showed that TPL2 overexpression was significantly positively correlated with low Ki67 positive index, low histological grade (grades 1–2) and early TNM stage (stage I) in IDC. It indicated that the malignancy of IDC with TPL2 overexpression is lower than that of IDC with low TPL2 expression, suggesting that patients with TPL2 overexpression may have a better prognosis than those with low TPL2 expression. However, as a molecule that promotes the development of IDC, why does TPL2 overexpression suggest a better prognosis for IDC patients? We speculate that TPL2 overexpression may promote the development of IDC in early stages, and its expression may decrease in late stages of tumor development due to the regulation of other signaling molecules. TPL2 overexpression may be an early molecular event in the development of IDC. With continued studies, TPL2 is expected to be a reliable indicator for monitoring the prognosis of breast cancer patients in the future.

Estrogen receptor (ER) is an important biomarker for guiding breast cancer treatment decisions. Endocrine therapy targeting ER can significantly improve the prognosis of breast cancer patients with ER positive expression. Thus, it is a common treatment strategy for such patients^28,29^. It has been reported that Pin1 can increase the ER protein level by inhibiting the proteasome-dependent degradation of the receptor^30^. Our data showed that the Pin1 overexpression rate of the ER positive expression group was significantly higher than that of the ER negative expression group in IDC, that is, the ER positive rate was higher in patients with Pin1 overexpression. These results indicate that patients with Pin1 overexpression are likely to respond to ER-targeted endocrine therapy and have a better prognosis.

In conclusion, TPL2 and Pin1 may synergistically promote the occurrence and development of IDC. However, TPL2 overexpression may only contribute to the early stages of IDC development. TPL2 overexpression is significantly associated with IDC with lower malignancy or earlier TNM stage, suggesting that IDC patients with TPL2 overexpression may have a better overall prognosis. Therefore, TPL2 is expected to be a reliable indicator of the prognosis of IDC patients.

## Conclusions

TPL2 and Pin1 may synergistically promote the occurrence and development of IDC, but TPL2 overexpression may be an early molecular event in IDC development. TPL2 overexpression is significantly associated with IDC with lower malignancy or earlier TNM stage, suggesting that the prognosis of IDC patients with TPL2 overexpression may be better. TPL2 overexpression may be a predictor of good prognosis in IDC, and the detection of the expression levels of TPL2 in IDC tissues in the future may be a key approach to evaluate the prognosis of IDC patients.

## Data Availability

The datasets generated and/or analysed during the current study are not publicly available due to confidentiality agreement but are available from the corresponding author on reasonable request.
